# Adoption of nonwire localisation devices in UK breast units: an iBRA-NET survey to assess changes in practice

**DOI:** 10.1308/rcsann.2024.0106

**Published:** 2025-07-24

**Authors:** F Mavor, SK Somasundaram, AR Carmichael, S Elgammal, R Foster, S Lowes, Y Masannat, R Milligan, JL Morgan, ER St John, R Vidya, RV Dave, J Harvey

**Affiliations:** ^1^ Manchester University NHS Foundation Trust, UK; ^2^ Royal Lancaster Infirmary, UK; ^3^ University Hospitals of Derby and Burton NHS Foundation Trust, UK; ^4^ NHS Ayrshire and Arran, UK; ^5^ Royal Liverpool and Broadgreen University Hospital, UK; ^6^ Newcastle University, UK; ^7^ Gateshead Health NHS Foundation Trust, UK; ^8^ Mid and South Essex Hospitals NHS Trust, UK; ^9^ University of Aberdeen, UK; ^10^ University of Sheffield Medical School, UK; ^11^ Portsmouth Hospitals University NHS Trust, UK; ^12^ University of Portsmouth, UK; ^13^ The Royal Wolverhampton NHS Trust, UK; ^14^ University of Manchester, UK

**Keywords:** Breast cancer, Localisation device, Magnetic seed, RFID tag, Radar reflector, Guidewire

## Abstract

**Introduction:**

A national practice questionnaire in 2020 collected data from UK breast surgeons on breast localisation device use, and found that wires were used most frequently. The current study aimed to assess the change in device use since the previous questionnaire, impact on logistics and clinician feedback.

**Methods:**

The 2020 national questionnaire was repeated, as well as adding qualitative questions to elicit themes important to clinicians in differentiating between the quality of localisation device experience. The electronic survey was distributed to UK breast surgeons and radiology members of the Association of Breast Surgery and British Society of Breast Radiology. The medians of the satisfaction responses for each device were compared with the median responses for wire.

**Results:**

There were 157 completed questionnaires, with 76 responses from surgeons and 81 from radiologists/radiographers, representing 84 UK breast units (August–December 2022). Localisation device use has changed; from 83% wire and 17% nonwire (5% radio-occult lesion localisation, 2% radioiodine seed and 9% Magseed) in 2020 to 18% wire and 82% nonwire (4% radioiodine seed, 49% Magseed, 6% SAVI SCOUT, 20% Hologic LOCalizer and 3% Sirius Pintuition) by 2022. In 2020 6% of patients had localisation performed before the day of surgery versus 65% by 2022 (*p*<0.05). Nonwire devices were preferred to wire in six themes for surgeons (*p*<0.05) and four for radiologists (*p*<0.05).

**Conclusions:**

UK breast surgery practice is shifting from use of guidewires towards newer localisation devices, with resultant logistical benefits and higher clinician satisfaction.

## Introduction

Many breast cancers are impalpable and require image-guided localisation before surgical excision. For decades the standard localisation device has been wires, inserted under either ultrasound or stereotactic guidance using local anaesthetic. However, because wires typically require insertion on the day of surgery, this can cause theatre delays and increased anxiety for patients. Furthermore, with wires protruding from the skin, there is the additional risk of discomfort and wire dislodgement. In recent years there has been a shift towards using wire-free localisation techniques that can be inserted in an outpatient setting well in advance of surgery, allowing decoupling of radiology and theatres on the day of surgery and reducing patient anxiety.

In 2020, the iBRA-NET Localisation Group carried out a National Practice Questionnaire (NPQ),^1^ which showed that wires were used by most centres, with only a small proportion using wire-free devices. As more data emerge on the use of different devices, and as new devices come to the market, a change in clinical practice is inevitable. Evaluating these data and sharing practices will help to establish the relative advantages and disadvantages of the different devices, which will help inform clinicians of which device might work best in their institution.

Early wire-free techniques such as radio-guided occult lesion localisation (ROLL) and radioactive 125I seed localisation (RSL) were developed in the late 1990s and early 2000s, but both techniques carry the burden of using radioactive materials.^2^

Newer nonradioactive wire-free devices have been developed, which mitigate this disadvantage and demonstrate similar safety and effectiveness to wires. These include paramagnetic seeds,^3^ magnetic seeds,^4^ radiofrequency identification (RFID) tags and radar reflectors.^5,6^

The first UK iBRA-NET NPQ (NPQ1) collected data on the techniques used for impalpable breast lesions in 130 UK breast units.^1,7^ The response of one surgeon per unit was requested to reflect each individual unit's practice. The questionnaire included quantitative data on device usage, logistical and demographic data, and perceived limitations of their current localisation technique (primarily wire). The respondents were asked whether they would consider changing localisation technique and what the potential barriers were to change.

The aim of the present study (NPQ2) was to evaluate current practice and to establish how device usage has changed since the previous study. It also aimed to assess qualitative feedback from respondents on their perceived satisfaction with each device. Furthermore, since the satisfaction and effectiveness of device usage is dependent upon the experience of both surgeons and radiologists, separate parallel surveys were devised for the two specialties.

## Methods

This study was intended primarily as a follow up to the initial NPQ,^1^ designed to assess quantitatively which localisation devices are currently being used in breast units in the UK, as well as qualitative outcomes of satisfaction with the different devices. The previous survey was aimed at breast surgeons, but in the current study, two separate specialty-adapted questionnaires were created, one for completion by breast surgeons and the other by breast radiologists/radiographers; although most devices are inserted by breast radiologists, some specialist radiographers are trained in localisation device insertion.

Respondents were asked demographic and logistical questions, as well as which device is currently used the most in their unit, in an identical format to the initial NPQ (Appendix 1).

The quantitative data represent the unit practice and, as such, one response per unit was included. Some questions were asked only to surgeons and the first complete response per unit was used. The only discrepancy between multiple respondents was in the estimation of unit volume per year.

Some questions were asked to both surgeons and radiologists. In the instance of multiple responses per unit, the first complete surgeon response from each unit was used, then the first complete radiologist response was used from units that were not represented by a surgeon. There were no discrepancies between individual quantitative responses from radiologists. The algorithm used to determine which response would be used when multiple responses were received for a unit is included in Appendix 2.

The differences between NPQ1 and this study were analysed using nonparametric tests using SPSS version 28.0.1.0(142).

Data were collected on which devices had been trialled (fewer than ten cases) or used (ten or more cases) by each responding clinician, including wires, ROLL, RSL, Magseed (paramagnetic seeds), SCOUT (radar reflectors), LOCalizer (RFID tags), Pintuition (permanently magnetic seeds) or a free text option for any other device. A Likert scale was used for respondents to rate their satisfaction from 1 to 10 on different themes regarding the devices that they had either trialled or used.

The questions for each specialty-adapted survey are listed in Appendix 1. Some questions were common to both surveys. Surgeons were asked questions about logistics (questions 7–10) that were not asked to radiologists. In the questionnaire for radiologists/radiographers, respondents were first asked to clarify their occupation: radiologist, radiographer or a free text option for “other”. They were asked to specify who performs the localisation in an ultrasound-guided or stereotactic insertion.

Both questionnaires were compiled using SurveyMonkey (San Mateo, CA, USA). A pilot version was completed by four consultant surgeons and four consultant radiologists from three breast units. Following feedback from the pilot, appropriate revisions were made before the survey going live.

The finalised questionnaires were circulated via the Association of Breast Surgery (ABS) newsletter, the British Society of Breast Radiology (BSBR) newsletter and social media. The questionnaires were completed online between August and December 2022.

For statistical analysis to be performed, at least ten responses for each device were required in all themes. Any devices not meeting that threshold were therefore excluded from statistical analysis. The median values of each of the satisfaction responses for the localisation devices that had received more than ten responses in all themes were compared with the median responses for wire as the standard. All the nonwire devices were also combined for a total nonwire result. Statistical significance was calculated using a Mann–Whitney *U* test using SPSS version 28.0.1.0(142).

## Results

### Unit responses: surgeons

The responses regarding logistics are summarised in Table 1. Fifty-one units were represented by surgeons (*n*=51).

**Table 1:** Summary of logistical data surgeon responses in NPQ 2 (*n*=51 units) compared with the unit responses to NPQ 1 (*n*=98 units)*

	NPQ 1 (*n*=98)	NPQ 2 (*n*=51)	χ^2^
Timing of first localisation-guided surgery on a morning list			
8am–9am	15 (15%)	33 (65%)	*p*<0.001
9am–10am	26 (27%)	12 (23%)	
10am–11am	42 (43%)	3 (6%)	
11am–12pm	11 (11%)	2 (4%)	
After 12pm	4 (4%)	1 (2%)	
Theatre delays due to waiting for patients after localisations			
Daily	6 (6%)	0 (0%)	*p*<0.001
Once or twice a week	33 (34%)	6 (12%)	
Rarely (once or twice a month)	44 (45%)	13 (25%)	
Never	15 (15%)	32 (63%)	
Theatre overruns due to waiting for patients after localisations			
Daily	1 (1%)	0 (0%)	*p*<0.001
Once or twice a week	15 (15%)	2 (4%)	
Rarely (once or twice a month)	48 (49%)	16 (31%)	
Never	34 (35%)	33 (65%)	

NPQ = National Practice Questionnaire.

*These questions were directed only to surgeons in NPQ 2 as per NPQ 1. One response per unit was included.

### Unit responses: surgeons and radiologists

Of the 130 breast units in the UK, 84 units were represented (*n*=84); 51 were represented by surgeons and 59 by radiologists and radiographers. The first complete surgeon response was used to represent the unit to allow for direct comparison with NPQ1, which surveyed only surgeons. In units that did not have complete surgeon responses, the radiologist response was used. Thirty-three unit responses from radiologists were included in the results.

**Figure 1:**
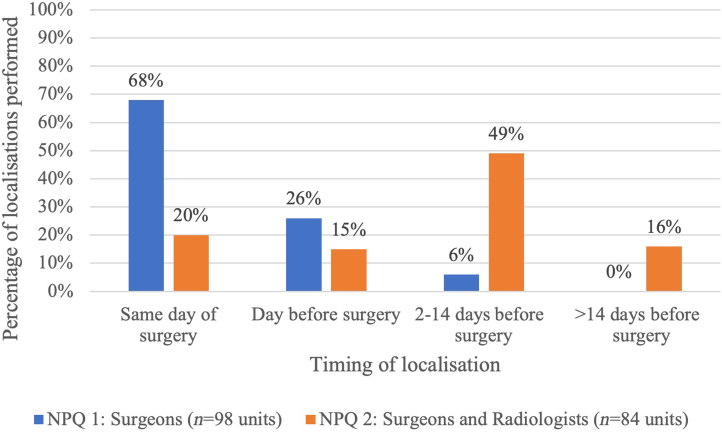
Timepoint in patient pathway that localisation takes place; comparing national practice in 2020 (NPQ1) with 2022 practice (NPQ2). This bar chart demonstrates the differences between practice in 2020 (blue) with those in 2022 (orange) (*p<0.001). NPQ = National Practice Questionnaire.

Figure 1 shows the timings of when patients have their preoperative localisation device inserted. Sixty-eight percent of patients had their device inserted on the day of surgery in the 2020 NPQ, which decreased to 20% in the 2022 NPQ. There is a significant difference between when patients in the first NPQ had their preoperative localisation when compared with this study (*p*<0.001).

The current localisation methods used in the UK based on the breast units that responded are represented in Table 2. In 2020, wires accounted for 83% of the localisation devices used, but in 2022 this decreased to 18%, with paramagnetic seeds now representing the most-used device, accounting for 49%. Several new devices are used in 2022 that were not used or available in 2020.

**Table 2:** Frequency of localisation device use for impalpable breast lesions in UK breast units*

	NPQ 1: surgeons (*n*=98 units)	NPQ 2: surgeons and radiologists (*n*=84 units)
Wire	82 (83%)	15 (18%)
ROLL	5 (5%)	0 (0%)
RSL	2 (2%)	3 (4%)
Magseed	9 (10%)	41 (49%)
SCOUT	0 (0%)	5 (6%)
LOCalizer	0 (0%)	17 (20%)
Pintuition	0 (0%)	3 (3%)

NPQ = National Practice Questionnaire; ROLL = radio-guided occult lesion localisation; RSL = radioactive ^125^I seed localisation.

*Data from NPQ1 (2020) and NPQ2 (2022) were compared. One response per unit was accepted. In instances where there was more than one response per unit, the first response was accepted.

### Individual responses

There were 157 complete individual responses in total: 76 complete responses from the survey for surgeons and 81 from the survey for radiologists (*n*=64), breast clinicians (*n*=5) and radiographers (*n*=12). The devices that had been trialled (fewer than ten cases) or used (ten or more cases) by individual breast surgeons and radiologists/radiographers are represented in Table 3.

**Table 3:** Localisation devices trialled or used by individual surgeons and radiologists/radiographers in the second NPQ of localisation devices for impalpable lesions*

	Devices trialled (fewer than ten cases)		Devices used (at least ten cases)	
	NPQ 2: surgeons (*n*=76)	NPQ 2: radiologists (*n*=81)	NPQ 2: surgeons (*n*=76)	NPQ 2: radiologists (*n*=81)
Wire	1	33	61	65
ROLL	0	1	5	1
RSL	4	5	4	5
Magseed	44	40	44	37
SCOUT	13	16	10	6
LOCalizer	14	33	12	22
Pintuition	4	4	4	3

NPQ = National Practice Questionnaire; ROLL = radio-guided occult lesion localisation; RSL = radioactive ^125^I seed localisation.

*Responses reflect the devices that individual surgeon and radiologists had trialled/used in the past five years in any breast unit in which they had worked.

The median values of the satisfaction scores for the localisation devices in each theme are shown in Table 4. Statistical analysis was performed on devices where all themes received at least ten responses. Results that differ significantly from wire as the standard are marked.

**Table 4:** Median values of satisfaction scores with localisation devices with more than ten responses for surgeons and radiologists, including the total of all nonwire devices^‡^

	Risk of device dislodging	Ease of detection during surgery	Signal focus	Ease of detection in a deep breast	Ability to judge distance from probe during surgery	Ease of logistics	Ease of bracketing of lesions	Overall satisfaction	
Median values of surgeons' results (1=poor, 10=excellent)									
Wire (*n*=51)	6	8	4	7	4	4	8	7	
Magseed (*n*=43)	9^*^	8.5	8^*^	6	7^**^	9^**^	7	8^**^	
ROLL (*n*=3)	6.5^†^	9^†^	9^†^	8^†^	6^†^	5^†^	5^†^	8^†^	
RSL (*n*=3)	9^†^	10^†^	10^†^	10^†^	8^†^	10^†^	8^†^	10^†^	
SCOUT (*n*=12)	8	8	7	6	8^**^	9^**^	7	8	
LOCalizer (*n*=10)	7.5	9	8	8	8^**^	9^**^	8	7	
Pintuition (*n*=4)	6^†^	9.5^†^	9.5^†^	8.5^†^	9.5^†^	10^†^	8^†^	8.5^†^	
Pooled nonwire (*n*=75)	8^*^	9^*^	8^*^	7	8^**^	9^**^	7	8^**^	
Median values of radiologists' results (1=poor, 10=excellent)									
Wire (*n*=50)	8.5	9	8	9	8	9	8	6	7
Magseed (*n*=32)	9^*^	9^*^	8^*^	9	9^**^	9	8^*^	9^**^	9^**^
ROLL (*n*=2)	8^†^	10^†^	10^†^	9.5^†^	10^†^	9^†^	10^†^	8^†^	8^†^
RSL (*n*=7)	10^†^	10^†^	8^†^	10^†^	8.5^†^	10^†^	8.5^†^	6^†^	9^†^
SCOUT (*n*=9)	9^†^	9^†^	8^†^	8^†^	8^†^	8^†^	8^†^	9^†^	8^†^
LOCalizer (*n*=23)	9	7.5^*^	7	8^*^	7	8^*^	7	10^**^	8
Pintuition (*n*=1)	8^†^	6^†^	6^†^	7^†^	6^†^	8^†^	6^†^	10^†^	7^†^
Pooled nonwire (*n*=74)	9^*^	9	8	9	8^*^	9	8	9^**^	9^**^

ROLL = radio-guided occult lesion localisation; RSL = radioactive ^125^I seed localisation.

^‡^Devices that did not receive at least ten responses in all themes were excluded from statistical analysis due to low power. Asterisks indicate where satisfaction differs significantly from wire as the standard (*p<0.05, **p<0.001). ^†^Results that were not analysed statistically due to fewer than ten responses.

**Figure 2:**
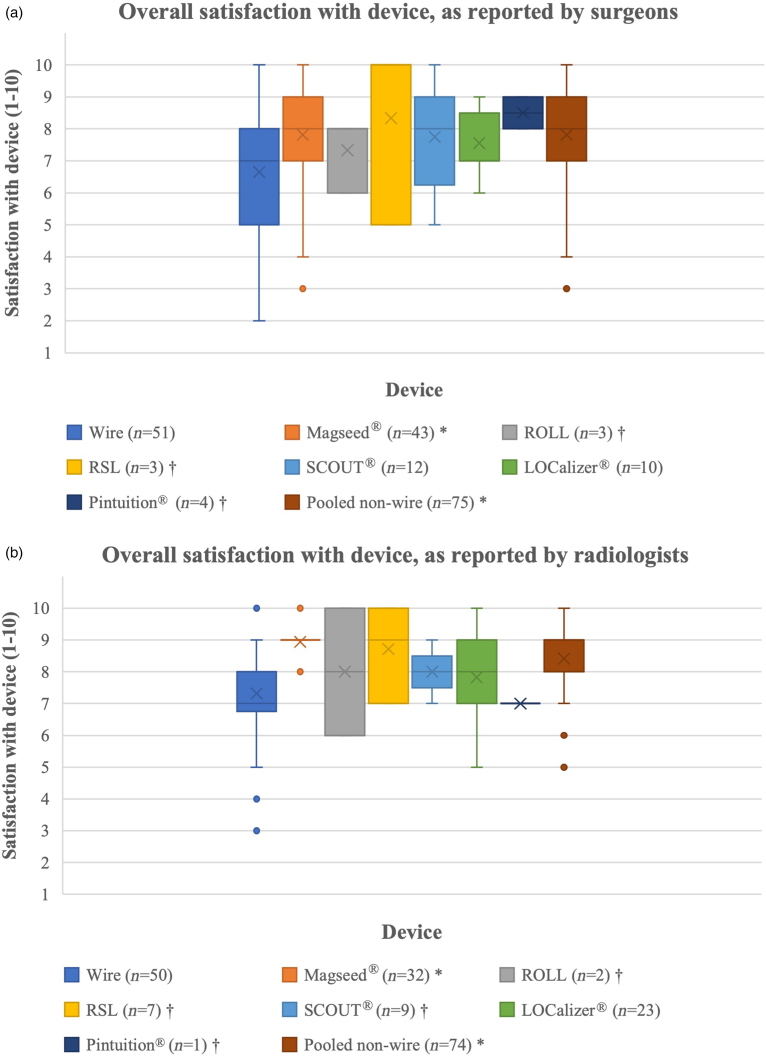
NPQ results for median overall satisfaction scores for each device and the total responses for all nonwire devices, as reported by (a) surgeons and (b) radiologists. No statistical analysis was performed on the devices with fewer than ten responses; 95% confidence intervals are included. *Results statistically significantly different to wire; ^†^results where statistics were not performed due to low number of responses. NPQ = National Practice Questionnaire; ROLL = radio-guided occult lesion localisation; RSL = radioactive 125I seed localisation.

For surgeons, when all the nonwire devices were combined, the results were preferable to wire in six themes (*p*<0.05). The satisfaction scores for Magseed were statistically significantly higher than for wire in five of eight themes (*p*<0.05). The satisfaction scores for SCOUT and LOCalizer were significantly higher than for wire in two themes each (*p*<0.001). The overall score for satisfaction with all of the devices from surgeons is shown in Figure 2a, including the devices that were excluded from statistical analysis due to receiving fewer than ten responses.

For radiologists, when all the nonwire devices were combined, the results were preferable to wire in four themes (*p*<0.05). The satisfaction scores for Magseed were statistically significantly higher than for wire in seven of nine themes (*p*<0.05). The satisfaction score for LOCalizer was significantly higher than for wire in one (*p*<0.001) theme: ease of logistics. The radiologists' satisfaction scores for SCOUT are not included in the results due to fewer than ten responses in some of the themes. The overall score for satisfaction with all the devices from radiologists is shown in Figure 2b, including the devices that were excluded from statistical analysis due to receiving fewer than ten responses.

No statistical analysis in terms of satisfaction for ROLL, RSL or Pintuition compared with wires could be carried out due to the small sample size of respondents who had trialled or used these devices (fewer than ten responses).

Another key consideration is any potential barriers to introducing a new nonwire service to replace guidewires: the survey findings suggest that most units have success in applying for a business case to support the new service (Appendix 3).

Responses not included in the main body of the results are included in Appendix 3.

## Discussion

This study highlights that localisation practice has changed significantly over the past few years, with the use of guidewires, previously the main localisation device, decreasing from 83% in 2020 to 18% in 2022. Based on the respondents in the current survey, the localisation device used most frequently in the UK is now paramagnetic seeds, accounting for 49% of units. As might be expected, the study also found an increase in the use of newer alternative techniques, such as RFID, magnetic seeds and radar reflectors. Radar reflectors and RFID were not CE marked at the time of the first study.

With time, it is expected that routine wire use will decline further as units continue to adopt wire-free techniques. The relative proportion of different device usage will also likely continue to change as different centres trial and adopt devices that work best for them, and as newer generations of devices come to the market, together with a growing literature on how these devices perform in clinical practice (Table 5). Based on the qualitative data in this study on the satisfaction of individual surgeons, radiologists and radiographers with each device, our findings indicate that wire-free devices are overall preferred to wires, supporting the likely continued trend towards making wires all but obsolete. The preference for nonwire devices over guidewires is multifactorial, with reported benefits including reduced risk of the device dislodging, easier detection during surgery and easier logistics, signal focus and better ability to judge distance from a probe.

**Table 5:** iBRA-net studies comparing wire-guided localisation and localisation devices: publications and prospective studies

Device	Study approval and funding	Recruitment	Data verification	Data analysis	Outcome	Results publication
Magseed	✓	✓	✓	✓	Magseed demonstrated equivalent safety and effectiveness to wire localisation	Dave *et al* 2022^3^
LOCalizer	✓	✓	✓	✓	LOCalizer demonstrated equivalent safety and effectiveness to wire localisation	Harvey *et al* 2024^8^
SCOUT	✓	✓	December 2024	February 2025		
Pintuition	✓	✓	January 2025	March 2025		

ROLL = radio-guided occult lesion localisation; RSL = radioactive 125I seed localisation.

The survey data indicate that each device has its own advantages and disadvantages, with none standing out above the others in excelling in all areas of use. This is to be expected given that each device works in a slightly different way, with multiple contributing variables to their usage, including but not limited to: device/introducer size, mode of detection and detector probe size. Even between the same devices, personal preference will still also play a role. The relatively small sample sizes for some of the devices is also likely to be a contributing factor, which is an accepted limitation of this study. A further qualitative analysis of the themes may enable direct comparison between all devices in a larger, perhaps multinational, study, although as newer devices and newer generations of the existing devices come to the market, this will change again.

Importantly, there has been a significant improvement in logistics associated with the adoption of wire-free techniques, creating more efficient clinical services and improving the patient pathway. In the 2020 NPQ, most patients had their preoperative localisations done on the day of surgery, reflecting the predominance of guidewires at the time, but now, with wire-free techniques predominating, most patients have their localisations done two or more days before surgery. This also coincides with earlier theatre start times for localisation-guided surgery, and fewer theatre delays and overruns due to patients waiting for their localisations. It would be expected that these benefits to the patient pathway would translate to a better patient experience, though this is something that has not yet been investigated across UK centres.

Studies of this type inevitably have their limitations, and the relatively small *n* values for certain devices is one potentially confounding factor in interpretation of the data. There is potential bias when using a survey to collect data as the responses often rely on perception. This bias is consistent with the bias of the previous survey in 2020, where the some of the same questions were asked to respondents. The perception of a change in practice with earlier start times has been reported by clinicians but in future studies it would be ideal to validate this perception with quantitative data. However, the reporting of earlier start times is unlikely to be due to bias as there was a very different response in this survey compared with when the same question was asked in 2020. Similarly, because this was a voluntary survey, not every breast unit is represented. This also introduces potential selection bias from respondents.

The first complete surgeon response was also taken to reflect an individual unit's practice (with the first complete radiologist responses taken in units without a surgical response) to compare with the first NPQ (which just included surgeons). This means that where the responses of surgeons and radiologists differed, only the surgical response was included.

## Conclusion

This follow-up survey highlights a change in practice from mainly wire localisation to nonwire devices. The benefits appear to be an ability to place devices in advance, earlier start times in theatre and higher clinician satisfaction.

The qualitative data show that clinicians differentiate between the qualities of localisation devices and demonstrate that nonwire devices are preferable to wire in several important themes. As other localisation devices become used more widely, future studies will be vital to evaluate clinicians' preferences to guide further change in practice and, importantly, take into account the patient's perspective.

## Author contributions

All authors contributed to the study conception and design. Material preparation, data collection and analysis were performed by F Mavor, J Harvey and R Dave. The first draft of the manuscript was written by F Mavor and all authors commented on previous versions of the manuscript. All authors read and approved the final manuscript.

## Conflicts of interest

The authors declare that no funds, grants, or other support were received during the preparation of this manuscript. F Mavor, S Somasundaram, A Carmichael, S Elgammal, J Morgan and R Vidya disclosed no conflicts of interest. S Lowes and R Milligan received standard class travel reimbursement and a speaker fee for a presentation on wire-free localisation techniques at a Hologic-sponsored event. Y Masannat is the owner and editor of iBreastBook and Endomag, merit, Hologic and pintuition all have sponsored educational events for iBreastBook. E St John received financial support from Sirius Medical for attending symposia and as project sponsor for the iBRA-NET Pintuition Localiser study. R Dave received bursary for travel from Endomag. J Harvey received funding for travel and expenses to attend symposia to present research data from Merit and Endomag.
